# Epilepsy and Its Treatment

**Published:** 1904-10

**Authors:** 


### Books and Magazines.

---

Epilepsy and its Treatment. By Wm. P. Spratling, M. D., Medical Superintendent of the Craig Colony for Epileptics; Secretary of the National Association for the Study of Epilepsy and the Care and Treatment of Epileptics; Member American Medico-Psychological Association; New York Academy of Medicine; Buffalo Academy of Medicine; Rochester Pathological Society, American Medical Association, etc. Fully illustrated.

Cloth, 500 pages, $4.00. W. B. Saunders & Co., Philadelphia, New York, London, 1904.

This book is most timely, and much needed to bring the subject of epilepsy and its proper treatment up abreast with the advancement and progress in the knowledge of the subject that has been gained by clinical observation and experiment in a large number of cases within the recent past. No work of authority on the subject has appeared in the United States since that of Echverria was published in 1870. This fact was one of the considerations which led Dr. Spratling to publish his book. That it will meet with a ready and large sale is assured, because there is a real want of more advanced information on the subject than is available; because, too, of Dr. Spratling's well-known ability in this, his special line, and because, further, greater interest in and attention to the study of epilepsy now obtains than ever before. Recent researches have thrown light upon the cause and pathology of this distressing malady, heretofore almost a *terra incognita,* and an opprobrium; its treatment being for the most part, heretofore, empyrical and unsuccessful. It is no longer "given up" as an incurable disease. D.

